# Association of D-dimer Levels in Acute Ischaemic Stroke Patients at Tertiary Care Centre in Northern India: A Prospective Observational Study

**DOI:** 10.7759/cureus.90313

**Published:** 2025-08-17

**Authors:** Virendra Atam, Manohar K Jha, Nikhil K Gupta, Isha Atam, Sagar Srivastava, Akashdeep Sharma

**Affiliations:** 1 Internal Medicine, King George's Medical University, Lucknow, IND; 2 Physiology, King George's Medical University, Lucknow, IND

**Keywords:** acute ischemic stroke, biomarkers, d-dimer, in-hospital mortality, modified rankin scale, nihss score, prognostic indicators, risk stratification, stroke severity, thrombotic burden

## Abstract

Background

D-dimer, a fibrin degradation product, has emerged as a potential biomarker in acute ischemic stroke (AIS) due to its association with thrombotic activity. This study aimed to evaluate the association between D-dimer levels and stroke severity, in-hospital outcomes, and functional status in AIS patients admitted to a tertiary care center.

Methods

This prospective observational study was conducted over one year and included 120 patients diagnosed with AIS. Demographic, clinical, and radiological data were recorded. Stroke severity was assessed using the National Institutes of Health Stroke Scale (NIHSS), while functional outcomes were evaluated with the modified Rankin Scale (mRS). D-dimer levels were measured on admission, and patients were followed through hospitalization. Statistical analyses included analysis of variance (ANOVA), t-tests, and Pearson correlation.

Results

The mean age of patients was 57.09±15.65 years, with a slight female predominance (62, 51.67%). Hypertension (87, 72.50%) and diabetes (44, 36.67%) were the most common comorbidities. Middle cerebral artery (MCA) territory stroke was the most frequent subtype (30, 25%), and 67 (55.83%) patients had severe stroke (NIHSS≥16). In-hospital mortality was observed in 36 (30%) cases, while 84 (70%) patients were discharged. Mean D-dimer levels increased with stroke severity: 46.30±79.72 mcg/dL in mild, 60.35±69.12 mcg/dL in moderate, and 195.84±268.98 mcg/dL in severe strokes (p<0.0001). Patients who died had significantly higher D-dimer levels (253.93±339.77 mcg/dL) compared to those discharged (84.14±103.76 mcg/dL; p=0.0043). D-dimer showed a strong positive correlation with NIHSS (r=0.873) and a moderate correlation with modified Rankin Scale (mRS) (r=0.367), both statistically significant.

Conclusion

Elevated D-dimer levels were significantly associated with stroke severity and poor in-hospital outcomes. These findings support the utility of D-dimer as a prognostic biomarker in AIS and suggest its potential role in early risk stratification.

## Introduction

Stroke, particularly acute ischemic stroke (AIS), remains a leading cause of morbidity and mortality worldwide. The World Health Organization (WHO) defined stroke in 1970 as “rapidly developing clinical signs of global or focal disturbance in the cerebral function that lasting more than 24 hours or leading to death with no notable cause other than of vascular origin.” Although widely used, this definition is now considered outdated due to advancements in stroke imaging, diagnostics, and understanding of stroke mimics. Global epidemiological data from 2019 revealed that 101.5 million individuals had experienced a stroke, with 77.2 million affected by ischaemic stroke specifically. The burden is particularly high in Southeast Asia, East Asia, the Middle East, and Oceania, accounting for millions of deaths and substantial years of life lost to disability [1-7].

Ischaemic stroke is primarily caused by thrombotic or embolic events that impair cerebral blood flow. The underlying etiology significantly influences the prognosis and long-term outcomes of patients [5]. Despite advances in acute management, stroke survivors often require ongoing assistance with daily activities, representing a major personal and healthcare system burden. In China, for example, the healthcare infrastructure faces intense pressure due to the high volume of stroke cases [5].

Recent efforts to improve stroke prognosis have focused on identifying blood-based biomarkers that reflect underlying pathophysiological mechanisms. One such marker is D-dimer, a fibrin degradation product released during clot dissolution. Owing to its long half-life and stability, D-dimer testing is already used in the diagnostic evaluation of thrombotic conditions like deep vein thrombosis and pulmonary embolism. Elevated D-dimer levels are indicative of increased fibrin turnover and systemic coagulation activation [6,7].

Several studies have explored the clinical utility of D-dimer in the context of acute ischemic stroke. Elevated D-dimer levels have been correlated with infarct volume, specific stroke subtypes, and worse clinical outcomes [8]. Whiteley et al. [9] and Abd-Elhamid et al. [10] demonstrated associations between high D-dimer and poor prognosis, while Zhang et al. [11] showed increased stroke risk with elevated D-dimer. Systematic reviews by Yuan et al. [12] and clinical studies by Choi et al. [13] have further highlighted D-dimer’s role in predicting recurrence and long-term outcomes after embolic strokes.

However, most of these studies were conducted in non-Indian populations and often lacked regional stratification, limiting their generalizability. Additionally, there is limited literature addressing the utility of D-dimer as a prognostic tool in acute ischemic stroke patients within the Indian healthcare setting. This gap in evidence, combined with regional variability in stroke risk profiles and healthcare access, underscores the need for localized research.

Hence, this study was designed to evaluate the association of D-dimer levels in AIS patients at a tertiary care centre in northern India, aiming to assess its clinical relevance in prognostication and outcome prediction.

## Materials and methods

The aim of the present study was to evaluate the association of D-dimer levels in AIS patients, with a specific focus on early identification of large vessel occlusion (LVO). This prospective observational study was conducted in the Department of Medicine, King George's Medical University, Lucknow, over a one-year period from 1 May 2024 to 30 April 2025.

Participants and sample size

A total of approximately 120 patients were enrolled in the study. The sample size was estimated using the formula:

$N = \frac{(Z_{1 - \alpha/2})^2 \cdot p \cdot (1 - p)}{d^2}$, where p=0.02, α=0.05, and d=0.08. The calculated minimum sample size was 120 participants.

Inclusion criteria

Patients were eligible if they were aged above 18 years, suspected to have acute stroke, and presented within six hours from symptom onset. Blood samples had to be collected prior to thrombolytic therapy. Written informed consent was a prerequisite for enrollment.

Exclusion criteria

Patients who did not provide consent or those in whom LVO was not assessed using computed tomography angiography (CTA), magnetic resonance angiography (MRA), or transcranial color-coded duplex were excluded from the study.

Study procedures and measurements

Upon admission, venous blood samples were collected into sodium citrate tubes and centrifuged at 1500 g for 15 min at 4°C. The separated plasma aliquots were immediately stored at −80°C until biomarker assays were performed. D-dimer measurements were conducted in duplicate to ensure reliability, maintaining a mean coefficient of variation of less than 20%.

Clinical and radiological data were recorded using standardized forms. Stroke severity was quantified using the National Institutes of Health Stroke Scale (NIHSS) [1], Glasgow Coma Scale (GCS) [2], and modified Rankin Scale (mRS) [3]. NIHSS, GCS, and mRS scales used in this study are open access. LVO assessment was performed using CTA, MRA, or transcranial color-coded duplex by neurologists or radiologists blinded to biomarker levels. LVO was defined as occlusion involving tandem lesions, the intracranial internal carotid artery, middle cerebral artery (M1 and M2 segments), or basilar artery. A sensitivity analysis excluding M2 segment occlusions was also performed. Cases without LVO included ischemic strokes or transient ischemic attacks without major artery occlusion, hemorrhagic strokes, or stroke mimics. All diagnoses were made by trained neurologists and confirmed by neuroimaging before biomarker data were unblinded.

This observational study has been reported as per Strengthening the Reporting of Observational Studies in Epidemiology (STROBE) guidelines.

Statistical analysis

Data were entered into Microsoft Excel (Microsoft Corp, Redmond, WA, USA) and analyzed using IBM SPSS Statistics, version 26 (IBM Corp., Armonk, NY, USA). Continuous variables were expressed as means and standard deviations. Categorical variables were presented as counts and percentages. The Chi-square test was used for categorical comparisons, while Student’s t-test was employed for comparing group means. Correlation analyses were conducted to assess relationships between variables. A p-value <0.05 was considered statistically significant. A power analysis was included during the sample size estimation to ensure adequate statistical power for detecting meaningful differences.

## Results

A total of 120 patients diagnosed with AIS were enrolled in the study. The mean age of the cohort was 57.09±15.65 years, with the majority falling in the 50-64 years (46, 38.33%) and 65-79 years (41, 34.17%) age groups. Female patients slightly outnumbered male patients (62 vs 58, 51.67% vs. 48.33%). The mean BMI was 26.66±4.41 kg/m². Among clinical risk factors, hypertension was most prevalent (87, 72.50%), followed by type 2 diabetes mellitus (44, 36.67%) and hyperlipidemia (40, 33.33%) (Table 1).

**Table 1:** Demographics and Risk Profile of Acute Ischemic Stroke Patients (n=120) Values are presented as mean ± standard deviation or number (percentage). BMI: Body mass index; HT: hypertension; DM: diabetes mellitus; ± in the first column indicates presence or absence of additional comorbidities.

Parameter	Value
A. Demographic Details	
Mean Age (years)	57.09±15.65
18-20	4 (3.33%)
20-34	9 (7.50%)
35-49	16 (13.33%)
50-64	46 (38.33%)
65-79	41 (34.17%)
80-94	4 (3.33%)
Male	58 (48.33%)
Female	62 (51.67%)
B. Anthropometric Profile	
Mean Height (cm)	164.65±14.10
Mean Weight (kg)	68.22±12.93
Mean BMI (kg/m²)	26.66±4.41
C. Clinical Risk Factors	
Hypertension	87 (72.50%)
Type 2 Diabetes	44 (36.67%)
Hyperlipidemia	40 (33.33%)
Smoking	26 (21.67%)
Alcohol Use	27 (22.50%)
D. Combined/Complex Risk Patterns	
Uncontrolled HT±Comorbidities	64 (53.30%)
Dyslipidemia±DM±HT	21 (17.50%)
Isolated HT	5 (4.20%)
Isolated Smoking/Alcohol Use	4 (3.30%)
Old Age Related	4 (3.30%)
Overweight+Dyslipidemia±DM	3 (2.50%)
No Risk Factor	21 (17.50%)

Regarding stroke type, the most common presentation was middle cerebral artery (MCA) territory stroke (30, 25%), followed by unspecified ischemic strokes (22, 18.33%) and left-sided strokes (16, 13.33%). According to NIHSS, the majority had severe stroke (NIHSS≥16; 67, 55.83%), with 41 (34.17%) patients presenting with moderate and 12 (10.00%) with mild stroke. On initial neurological evaluation, 27 (22.50%) had the best GCS score (E4V5M6), while 10 (8.33%) showed severely impaired consciousness (E1). By day 3, 27 (22.50%) still had the best GCS score, but seven (5.83%) remained severely impaired (Table 2).

**Table 2:** Stroke Characteristics and Neurological Scores in Patients with Acute Ischemic Stroke (n = 120) MCA: Middle cerebral artery; ACA: anterior cerebral artery; NIHSS: National Institutes of Health Stroke Scale; GCS: Glasgow Coma Scale. "E4V5M6" represents the highest possible GCS score; "E1" indicates severe neurological impairment.

Category	Frequency (n)	Percentage (%)
A. Stroke Type and Location		
MCA Territory Stroke	30	25.00
Unspecified Ischemic Stroke	22	18.33
Left-Sided Stroke	16	13.33
Right-Sided Stroke	12	10.00
ACA/Cerebellar Stroke	10	8.33
Stroke with Complications	6	5.00
Recurrent/Multiple Infarcts	5	4.17
Stroke with Altered Sensorium	6	5.00
Others/Unclassified	13	10.83
B. Stroke Severity (NIHSS Score)		
Mild Stroke (NIHSS < 8)	12	10.00
Moderate Stroke (NIHSS 8–15)	41	34.17
Severe Stroke (NIHSS ≥ 16)	67	55.83
C. GCS Assessment Over Days		
GCS Day 1: Best (E4V5M6)	27	22.50
GCS Day 1: Good (E4 others)	28	23.33
GCS Day 1: Severe (E1)	10	8.33
GCS Day 2: Best (E4V5M6)	26	21.67
GCS Day 3: Best (E4V5M6)	27	22.50
GCS Day 3: Severe (E1)	7	5.83

Functional outcomes assessed via the mRS showed that 36 (30%) patients had died (mRS=6), while 32 (26.67%) had no significant disability (mRS=2) and 16 (13.33%) were symptom-free (mRS=1). Clinical discharge rate was 84 (70%) (Table 3).

Biochemically, patients had a mean hemoglobin of 11.30±2.21 g/dL, total leukocyte count of 11,995.62±4,983.29/mm³, and platelet count of 167,100±96,047.90/mm³. Liver function tests and coagulation markers were moderately elevated, with mean serum glutamic-oxaloacetic transaminase (SGOT) at 63.20±57.12 U/L, serum glutamic-pyruvic transaminase (SGPT) at 59.69±62.45 U/L, and international normalized ratio (INR) at 1.74±0.50 (Table 3).

**Table 3:** Functional Outcome and Biochemical Profile of Patients with Acute Ischemic Stroke (n = 120) mRS: Modified Rankin Scale; SGOT: serum glutamic-oxaloacetic transaminase; SGPT: serum glutamic-pyruvic transaminase; HbA1c: glycated hemoglobin. Values are expressed as mean±standard deviation or number (percentage).

Parameter	Value
A. Modified Rankin Scale (mRS)	
mRS 1 - No symptoms	16 (13.33%)
mRS 2 - No significant disability	32 (26.67%)
mRS 3 - Slight disability	17 (14.17%)
mRS 4 - Moderate disability	13 (10.83%)
mRS 5 - Severe disability	6 (5.00%)
mRS 6 - Death	36 (30.00%)
B. Clinical Outcome Status	
Discharged	84 (70.00%)
Death	36 (30.00%)
C. Biochemical Parameters	
Hemoglobin (g/dL)	11.30±2.21
Total Leukocyte Count (/mm³)	11,995.62±4,983.29
Platelet Count (/mm³)	167,100±96,047.90
Total Bilirubin (mg/dL)	1.27±1.97
Direct Bilirubin (mg/dL)	0.69±1.35
SGOT (U/L)	63.20±57.12
SGPT (U/L)	59.69±62.45
HbA1c (%)	6.78±2.05
Serum Sodium (mEq/L)	137.22±8.69
Serum Potassium (mEq/L)	3.94±0.68
Prothrombin Time (PT) (s)	17.26±4.00
International Normalized Ratio (INR)	1.74±0.50

**Figure 1:**
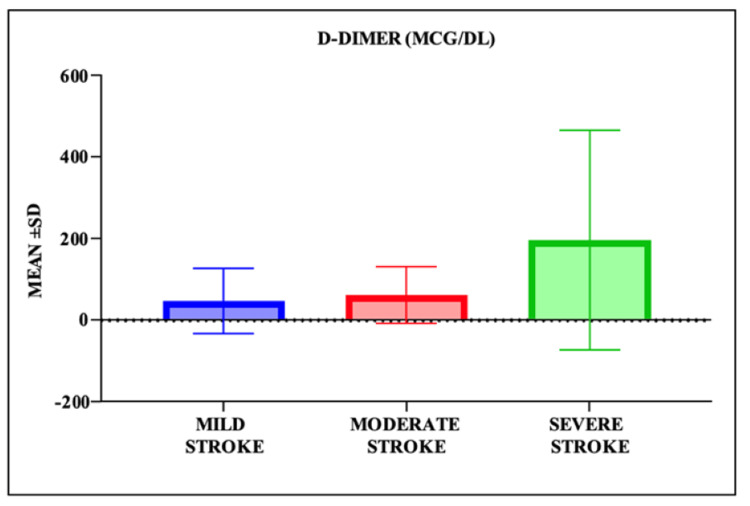
Mean D-Dimer Levels (mcg/dL) According to Stroke Severity (NIHSS Categories) Bar graph illustrating the mean±standard deviation (SD) of D-dimer levels in patients with mild (NIHSS<8), moderate (NIHSS 8–15), and severe (NIHSS≥16) acute ischemic stroke. A statistically significant increase in D-dimer levels was observed with increasing stroke severity (analysis of variance (ANOVA), F=29.42, p<0.0001). NIHSS: National Institutes of Health Stroke Scale.

Mean D-dimer levels progressively increased with stroke severity: 46.30±79.72 mcg/dL in mild, 60.35±69.12 mcg/dL in moderate, and 195.84±268.98 mcg/dL in severe stroke groups. This trend was statistically significant (analysis of variance (ANOVA), F=29.42, p<0.0001), as shown in Table 4 and visually depicted in Figure 1.

**Table 4:** Association Between D-Dimer Levels and Stroke Severity, Outcome, and Prognostic Scores (n=120) NIHSS: National Institutes of Health Stroke Scale; mRS: modified Rankin Scale; SD: standard deviation; t: Student’s t-test value

Outcome Group	D-dimer (mcg/dL, mean ± SD)	mRS (mean ± SD)	NIHSS (mean ± SD)	t-value	p-value
Expired (Deceased)	253.93±339.77	6.00±0.00	25.53±3.05	t=2.946	p=0.0043
Discharged	84.14±103.76	2.54±1.18	14.13±6.79		

**Figure 2:**
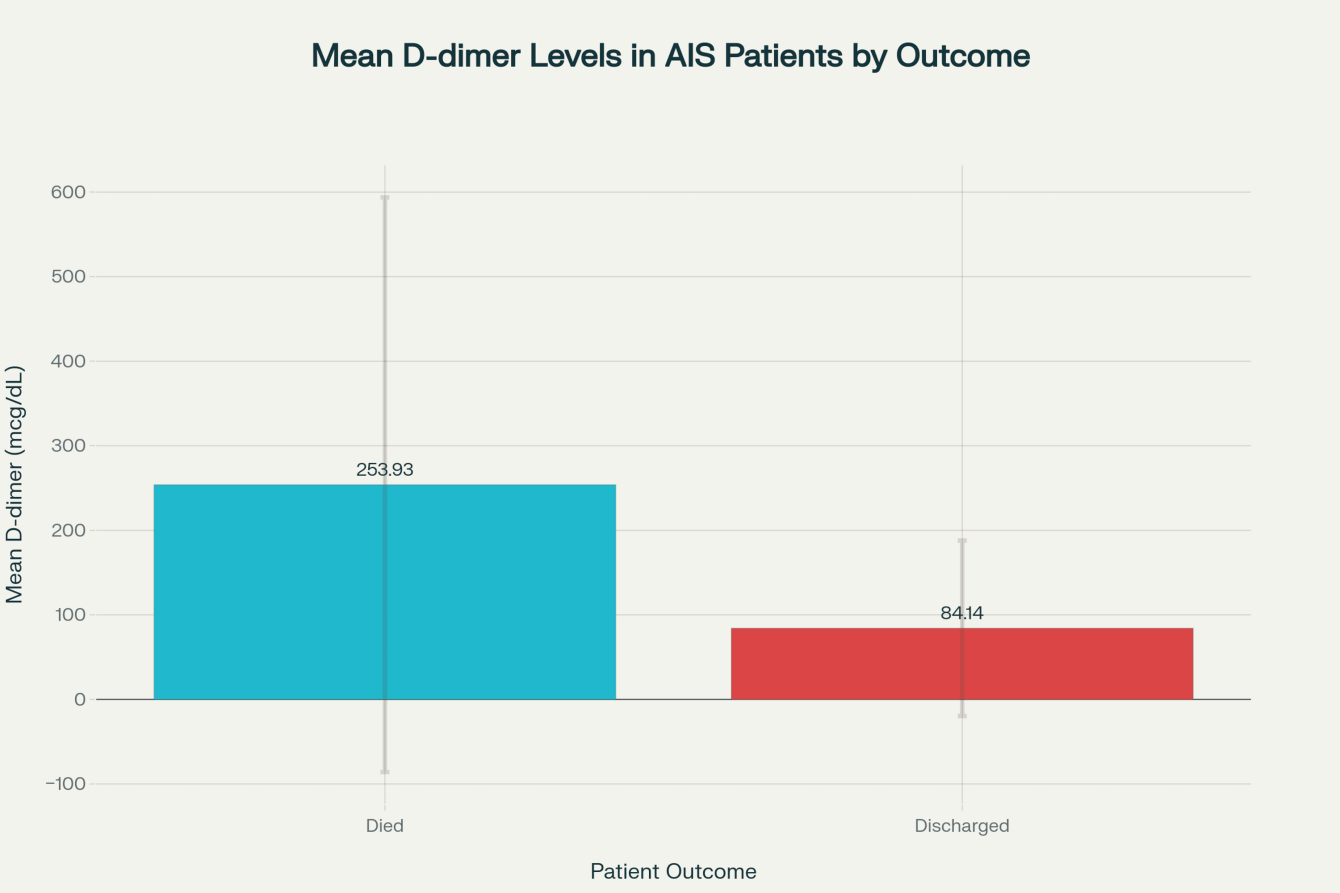
Comparison of D-Dimer Levels Between Expired and Discharged Patients Bar graph showing mean±standard deviation (SD) for D-dimer stratified by patient outcome. Patients who died had significantly higher D-dimer levels compared to those who were discharged.

A significantly higher mean D-dimer level was found in patients who died (253.93±339.77 mcg/dL) compared to those discharged (84.14±103.76 mcg/dL; t=2.946, p=0.0043). Correspondingly, the mRS and NIHSS scores were also higher among the expired group (mRS: 6.00±0.00; NIHSS: 25.53±3.05) than in discharged patients (mRS: 2.54±1.18; NIHSS: 14.13±6.79) (Table 4, Figure 2).

Pearson correlation analyses showed that D-dimer levels positively correlated with both NIHSS score (r=0.873, p=0.0001) and mRS (r=0.367, p=0.0001), suggesting that higher D-dimer concentrations were associated with greater stroke severity and worse functional outcomes (Table 5).

**Table 5:** Correlation Analyses NIHSS: National Institutes of Health Stroke Scale; mRS: modified Rankin Scale; r: Pearson’s correlation coefficient

Variable	Correlation Coefficient (r)	p-value
D-dimer vs NIHSS score	0.873	0.0001
D-dimer vs mRS	0.367	0.0001

## Discussion

This prospective observational study aimed to evaluate the association of D-dimer levels with stroke severity and outcome in patients with AIS. The mean age of the study population (57.09±15.65 years) reflected the middle-aged to elderly demographic commonly affected by cerebrovascular events, aligning with global estimates reported by Johnston et al. [4]. The slightly higher prevalence among women (62, 51.67%) is consistent with some regional stroke registries, although sex differences remain variable depending on age and geography [5].

Hypertension emerged as the most prevalent comorbidity (87, 72.50%), followed by diabetes (44, 36.67%) and hyperlipidemia (40, 33.33%). These findings reaffirm the dominant role of vascular risk factors in the pathogenesis of ischemic stroke, as emphasized in multiple epidemiological studies [6].

The hematological analysis of stroke patients revealed a mean hemoglobin level of 11.30±2.21 g/dL, indicating a high prevalence of anemia within the cohort. Anemia in stroke patients may worsen cerebral hypoxia and negatively affect neurological recovery. The total leukocyte count was elevated (11995.62±4983.29/mm³), reflecting an active inflammatory or stress response commonly associated with acute ischemic events. Meanwhile, the mean platelet count was 16,7100±96047.90/mm³, remaining within a broadly normal range but demonstrating considerable variability, which may reflect individual differences in vascular or systemic stress during stroke. Similarly, Nayak et al. [14] reported a significant reduction in haemoglobin levels, particularly in expired patients, indicating a link between anemia and poor prognosis.

The mean HbA1c was 6.78±2.05%, suggesting a considerable proportion of patients had underlying disturbances in glucose metabolism, including prediabetes or diabetes, which are known risk factors for stroke. Furthermore, our HbA1c findings are supported by Wu et al. [15], who demonstrated that elevated HbA1c levels are associated with increased all-cause mortality during the first year following an acute ischemic stroke.

MCA territory infarction was the most frequent stroke subtype (30, 25%), in line with the predominance of anterior circulation strokes [7]. More than half of the patients (67, 55.83%) presented with severe neurological deficits (NIHSS ≥ 16), which may reflect delayed hospital presentation, extensive infarcts, or comorbidities, as noted by Ntaios [8].

Functional outcomes showed that 36 (30%) patients died during hospitalization. mRS and NIHSS scores at discharge remained significantly higher in non-survivors, reinforcing their predictive value for mortality and disability, as demonstrated in previous reports [9]. Similarly, in the Nayak et al. [14] study of 17 patients, 76.5% (13/17) survived and were discharged, while 23.5% (4/17) died. Essa et al. [16] reported a mortality rate of 25%.

Our results demonstrated that D-dimer levels significantly increased with stroke severity, from 46.30 mcg/dL in mild to 195.84 mcg/dL in severe strokes (p<0.0001), consistent with findings by Abd-Elhamid et al. [10] and Park et al. [17], who correlated D-dimer levels with infarct burden.

Additionally, non-survivors had markedly elevated D-dimer levels (253.93±339.77 mcg/dL) compared to discharged patients (84.14±103.76 mcg/dL), supporting previous studies by Zhang et al. [11] and Yao et al. [18] showing D-dimer as a predictor of poor prognosis. Correlation analysis showed a strong positive association between D-dimer and NIHSS (r=0.873), and a moderate association with mRS (r=0.367), further establishing its relevance.

Recent meta-analyses have echoed these findings. Yuan et al. [12] confirmed elevated D-dimer as a predictor of stroke risk and unfavorable outcome, while Choi et al. [13] identified a link with recurrence in ESUS patients.

Despite these strengths, the study had limitations. It was single-center, and imaging protocols were heterogeneous. Serial D-dimer measurements and long-term follow-up were not performed, which may limit dynamic prognostication. Additionally, confounding variables such as atrial fibrillation or malignancy were not uniformly excluded.

Taken together, our results reinforce the clinical relevance of D-dimer not only as a diagnostic aid but also as a prognostic biomarker. Its incorporation into early assessment protocols could aid risk stratification and therapeutic decision-making, particularly in embolic or cryptogenic stroke subtypes.

## Conclusions

This study demonstrated a significant association between elevated D-dimer levels and both stroke severity and poor clinical outcomes in patients with acute ischemic stroke. D-dimer levels were markedly higher in patients who presented with severe neurological deficits and in those who did not survive hospitalization. Strong positive correlations with NIHSS and mRS scores further supported the utility of D-dimer as a prognostic biomarker. Incorporating D-dimer assessment into early stroke evaluation may have enhanced risk stratification and guided clinical decision-making, particularly in resource-limited settings.
